# Supplementary figures and images for: miR-221-5p and miR-186-5p Are the Critical Bladder Cancer Derived Exosomal miRNAs in Natural Killer Cell Dysfunction
Source: Int J Mol Sci. 2022 Dec 2;23(23):15177. doi: 10.3390/ijms232315177 (PMC9740765; doi:10.3390/ijms232315177)

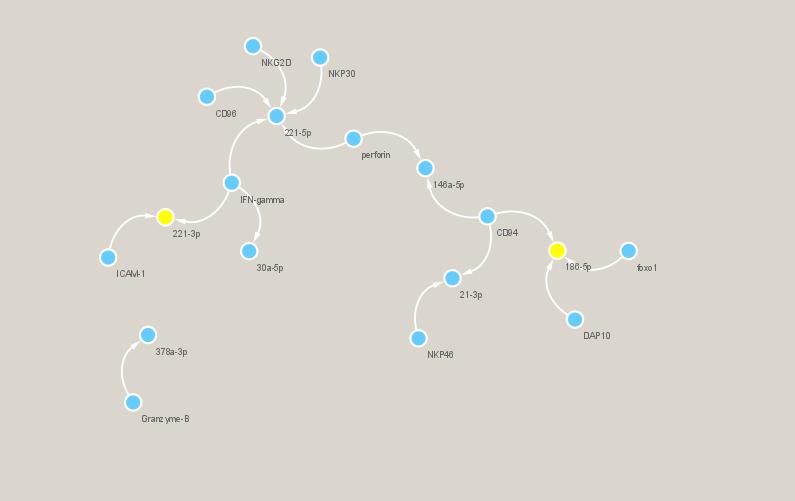

Supplement: Supplementary file 1 [file ijms-23-15177-s001.zip › Supplementary S1.jpeg]
